# Sequencing and analysis of the complete mitochondrial genome of flat-skulled shrew (*Sorex roboratus*) from China

**DOI:** 10.1080/23802359.2017.1334517

**Published:** 2017-07-04

**Authors:** Liu Zhu, Wang Ao-Nan, Zhang Jun-Sheng, Yang Xi, Liu Huan, Jin Zhi-Min

**Affiliations:** a College of Life Science and Technology, Mudanjiang Normal University, Mudanjiang, P.R. China;; b College of Wildlife Resources, Northeast Forestry University, Harbin, P.R. China

**Keywords:** Control region, mitogenome, phylogenetic trees, flat-skulled shrew

## Abstract

The complete mitogenome sequence of flat-skulled shrew (*Sorex roboratus*) was determined using long PCR. The genome was 17,153 bp in length and contained 13 protein-coding genes, 2 ribosomal RNA genes, 22 transfer RNA genes, 1 origin of L strand replication and 1 control region. The overall base composition of the heavy strand is A (33.1%), C (24.4%), T (29.4%), and G (13.1%). The base compositions present clearly the A–T skew, which is most obviously in the control region and protein-coding genes. The extended termination-associated sequence domain, the central conserved domain and the conserved sequence block domain are defined in the mitochondrial genome control region of flat-skulled shrew. Mitochondrial genome analyses based on MP, ML, NJ and Bayesian analyses yielded identical phylogenetic trees. The five *Sorex* species formed a monophyletic group with the high bootstrap value (100%) in all examinations.

In this paper, the complete mitochondrial genome of flat-skulled shrew (*Sorex roboratus*) was sequenced for the first time on ABI 3730XL using a primer walking strategy and the long and accurate PCR, with five pairs of long PCR primers and with 14 pairs of sub-PCR primers. A muscle sample was obtained from a female flat-skulled shrew captured from Huzhong region of Great Khingan Mountains in Heilongjiang Province, China (51°44′14″ N, 123°40′44″ E).

The mitochondrial genome is a circular double-stranded DNA sequence that is 17,153 bp long including 13 protein-coding genes, 2 rRNA genes, 22 tRNA genes, 1 origin of L strand replication and 1 control region. The accurate annotated mitochondrial genome sequence was submitted to GenBank with accession number KY930906. The arrangement of the multiple genes is in line with other Soricidae species (Nikaido et al. 2001; Fontanillas et al. 2005; Huang et al. 2014, 2016; Xu et al. 2016) and most mammals (Meganathan et al. 2012; Xu et al. 2012, 2013; Yoon et al. 2013).

The control region of flat-skulled shrew mitochondrial genome was located between the tRNA-Pro and tRNA-Phe genes, and contains only promoters and regulatory sequences for replication and transcription, but no structural genes. Three domains were defined in flat-skulled shrew mitochondrial genome control region (Zhang et al. 2009): the extended termination-associated sequence (ETAS) domain, the central conserved domain (CD) and the conserved sequence block (CSB) domain. Three CSBs were found in the CSB domain and they were located in positions 16,425–16,449, 16,865–16,895 and 16,918–16,943. Also only one repetitive sequence region (RS) was found, which was located between the CSB1 and CSB2, and was rich in A and C. The repetitive pattern of segments in the RS was 5′-TA-(TAC(T)ACG)n-TA-3′ (*n* = 46).

The total length of the protein-coding gene sequences was 11,428 bp. Most protein-coding genes initiate with ATG except for ND2, ND3 and ND5, which began with ATT or ATA. Seven protein-coding genes terminated with TAA whereas the Cyt b gene terminated with AGG. The incomplete stop codons (T– – or TA–) were used in ND1, ND2, COX3, ND3 and ND4. A strong bias against A at the third codon position was observed in the protein-coding genes. The frequencies of CTA (Leu), ATT (Ile), TTA (Leu) and ATA (Met) were higher than those of other codons. The length of tRNA genes varied from 59 to 75 bp. Twenty-one of them could be folded into the typical cloverleaf secondary structure except the tRNA-Ser (AGY), whose complete dihydrouridine arm was lacking.

Most flat-skulled shrew mitochondrial genes were encoded on the H strand, except for the ND6 gene and eight tRNA genes, which were encoded on the L strand. Some reading frame intervals and overlaps were found. One of the most typical was between ATP8 and ATP6. The L-strand replication origin (OL) was located within the WANCY region containing five tRNA genes (tRNATrp, tRNA-Ala, tRNA-Asn, tRNA-Cys, tRNA-Tyr). This region was 33 bp long and had the potential to fold into a stable stem-loop secondary structure. The total base composition of flat-skulled shrew mitochondrial genome was A (33.1%), C (24.4%), T (29.4%) and G (13.1%). The base compositions clearly present the A–T skew, which was most obviously in the control region and protein-coding genes.

In order to explore the evolution of Insectivora shrews which include Soricidae and Talpidae, especially the evolution of genus Sorex from China, here, we investigate the molecular phylogenetics of Chinese flat-skulled shrew using complete mitochondrial genome sequence of 26 species. All sequences generated in this study have been deposited in the GenBank (Figure 1).

**Figure 1.**
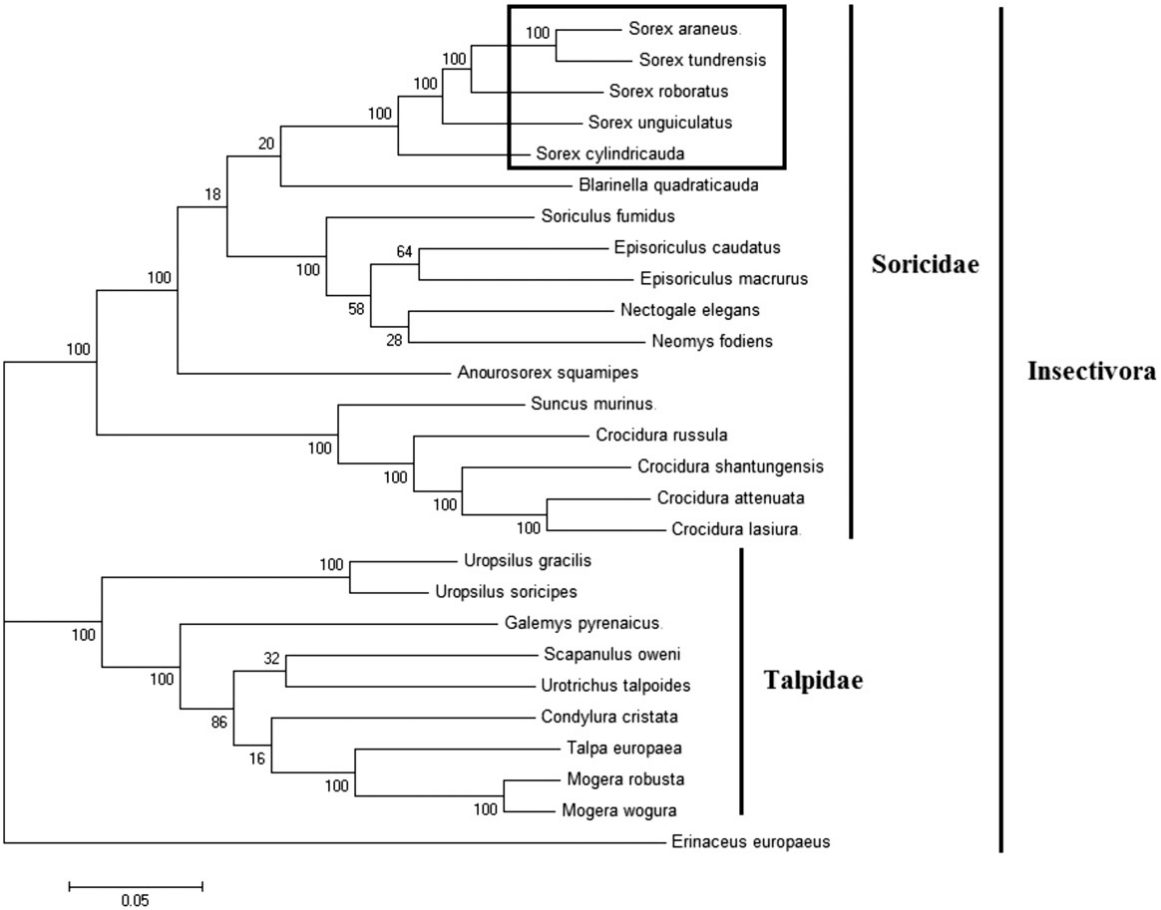
Phylogenetic tree generated using the Maximum Parsimony method based on complete mitochondrial genomes. *Crocidura lasiura* (KR007669), *Crocidura shantungensis* (JX968507), *Crocidura attenuata* (KP120863), *Crocidura russula* (AY769264), *Episoriculus macrurus* (KU246040), *Episoriculus caudatus* (KM503097), *Neomys fodiens* (KM092492), *Nectogale elegans* (KC503902), *Anourosorex squamipes* (KJ545899), *Blarinella quadraticauda* (KJ131179), *Suncus murinus* (KJ920198), *Soriculus fumidus* (AF348081), *Sorex araneus* (KT210896), *Sorex cylindricauda* (KF696672), *Sorex unguiculatus* (AB061527), *Sorex tundrensis* (KM067275), *Sorex roboratus* (KY930906), *Talpa europaea* (Y19192), *Urotrichus talpoides* (AB099483), *Uropsilus soricipes* (JQ658979), *Uropsilus gracilis* (KM379136), *Mogera wogura* (AB099482), *Mogera robusta* (KT934322), *Condylura cristata* (KU144678), *Galemys pyrenaicus* (AY833419), *Scapanulus oweni* (KM506754), *Erinaceus europaeus* (NC002080).

Mitochondrial genome analyses based on MP, ML, NJ and Bayesian analyses yielded identical phylogenetic trees, indicating a close phylogenetic affinity of shrews. The phylogram obtained from Maximum Parsimony method is shown in Figure 1. It shows that two major phyletic lineages were present in Insectivora: Soricidae and Talpidae. Soricidae comprised *Crocidura lasiura*, *Crocidura shantungensis*, *Crocidura attenuata*, *Crocidura russula*, *Episoriculus macrurus*, *Episoriculus caudatus*, *Neomys fodiens*, *Nectogale elegans*, *Anourosorex squamipes*, *Blarinella quadraticauda*, *Soriculus fumidus*, *Suncus murinus*, *Sorex araneus*, *Sorex tundrensis*, *Sorex roboratus*, *Sorex cylindricauda* and *Sorex unguiculatus* and was supported by bootstrap values of 100%. Talpidae comprised *Talpa europaea, Urotrichus talpoides, Mogera wogura, Condylura cristata, Uropsilus soricipes, Mogera robusta, Galemys pyrenaicus, Uropsilus gracilis and Scapanulus oweni* and was supported by bootstrap values of 100%. The three Sorex species formed a monophyletic group with the high bootstrap value (100%) in all the examinations.
